# Association between metabolic scores for visceral fat and chronic kidney disease: A cross-sectional study

**DOI:** 10.3389/fendo.2022.1052736

**Published:** 2022-12-05

**Authors:** Peng Yu, Xiaoyu Meng, Ranran Kan, Zhihan Wang, Xuefeng Yu

**Affiliations:** ^1^ Department of Endocrinology, Tongji Hospital, Huazhong University of Science and Technology, Wuhan, Hubei, China; ^2^ Branch of National Clinical Research Center for Metabolic Diseases, Wuhan, Hubei, China

**Keywords:** CKD, central obesity, anthropometric measurement, METS-VF, predictor

## Abstract

**Introduction:**

Central obesity is closely linked to the risk of chronic kidney disease (CKD). This study aimed to evaluate the association between the novel central obesity index- metabolic score for visceral fat (METS-VF) and the risk of CKD in a Chinese population, and to compare its ability to predict CKD with other central obesity indices including waist circumference (WC), waist-to-height ratio (WHtR), lipid accumulation product (LAP), visceral adiposity index (VAI), a body shape index (ABSI), body roundness index (BRI), and cardiometabolic index (CMI).

**Methods:**

This cross-sectional study included 8866 individuals from China. Demographic information, lifestyle data, and medical history data were collected, and physical examinations, anthropometric measurements and laboratory tests were performed for each participant. CKD was defined as an estimated GFR< 60 ml/min/1.73m^2^. Multivariate logistic regression models were used to evaluate the association between the METS-VF and the prevalence of CKD. Receiver operating characteristic (ROC) analyses were performed to assess and compare the predictive abilities of the central obesity indices and determine the optimal cut-off points.

**Results:**

A graded increase in the prevalence of CKD was observed with increasing METS-VF tertiles. Moreover, the METS-VF was independently associated with the risk of CKD after adjustment for current smoking, current drinking, physical activity level, diabetes, hypertension, CVD history and BMI. Compared with participants with a METS-VF in the lowest tertile, the multivariate-adjusted ORs and 95% CIs for participants with a METS-VF in the highest tertile were 3.943 (2.435-6.385) in the overall population, 3.585 (1.585-8.109) for men and 4.627 (2.485-8.616) for women. Significant interactions were found between the METS-VF and the risk of CKD by age (P value for interaction = 0.023). In ROC analysis, the METS-VF had a higher AUC value than other indices for predicting CKD in men and had comparable or higher AUC than other indices for women. For predicting CKD, the optimal cut-off value of the METS-VF was 6.891 for men and 6.744 for women. The METS-VF yielded the greatest Youden index among all indices for both sexes.

**Conclusion:**

A higher METS-VF was independently associated with a greater risk of CKD. The METS-VF can be a useful clinical indicator for identifying CKD, as it had superior predictive power for CKD when compared with other central obesity indices.

## Introduction

Chronic kidney disease (CKD) represents an enormous public health burden affecting 9.1% of the world’s population (1). It is defined as an estimated glomerular filtration rate (eGFR) less than 60 ml/min per 1.73m^2^ or demonstrated by markers of kidney damage that persist for at least 3 months. Approximately 2% of patients with CKD may progress into end-stage kidney disease (ESKD) (2). Moreover, the presence of impaired kidney function appears to have a marked impact on the risk of cardiovascular disease and its related mortality, and even a mild reduction in kidney function may have an adverse effect on cardiovascular health (3–5). Thus, the risk assessment of CKD in the general population is extremely important.

Obesity is a recognized risk factor for CKD. According to the 2011-2014 American National Health and Nutrition Examination Survey (NHANES), among individuals with CKD, 44% had obesity, 69% had elevated waist circumference, and the incidence rate of CKD paralleled the prevalence of obesity (6). Moreover, a global, collaborative meta-analysis that included more than five million individuals in 63 cohorts demonstrated that excessive adiposity is an independent risk factor for GFR decline (7). As a widely and frequently used index in obesity assessment, BMI has been most widely studied when assessing the relationship between obesity and CKD. For example, in the Framingham Offspring cohort, BMI was shown to be independently associated with the risk of CKD; for each 1 SD increase in BMI, a relative increase of 23.0% in the risk of CKD was observed (8). However, BMI has several limitations when assessing adiposity. First, BMI does not distinguish between lean and fat body mass, while sarcopenic obesity is highly prevalent among patients with CKD (9); thus, BMI may misclassify weight status among CKD patients. For example, the study performed by Dierkes et al. showed that 27.9% of the study participants were obese when using the BMI definition, while 48.8% of the study participants were obese when using the definition based on body fat percentage (which was measured by bioimpedance method) (10). Moreover, current studies indicate that the deleterious effect of obesity on kidney function is mainly attributed to excess visceral adiposity (11), whereas BMI is an index for overall obesity. Thus, indices that can provide accurate measurements of visceral obesity may be more helpful when assessing CKD risk, which is supported by a number of studies (12–16). For example, Oh et al. reported that central obesity indices such as WC, waist-to-hip ratio (WHR) and WHtR, but not BMI, were associated with the future risk of renal function decline (16).

The metabolic score for visceral fat (METS-VF) is a novel index for visceral adiposity, which was developed by nonlinear fits of an insulin resistance component (METS-IR), waist-to-height ratio (WHtR), age, and sex by using dual X-ray absorptiometry (DXA) as the reference. It has been validated by magnetic resonance imaging (MRI) and bioelectrical impedance analysis (BIA), which were used to measure visceral adipose tissue mass in an external population, and showed superiority over several other surrogate indices of visceral adiposity (17). However, the link between the METS-VF and the risk of CKD is still unknown. In this cross-sectional study, we therefore examined this association. At the same time, we aimed to compare the predictive ability of the METS-VF with other visceral adiposity indices, including WC, WHtR, LAP, VAI, ABSI, BRI, and CMI, for detecting CKD. This may help to determine the most appropriate visceral adiposity index for CKD risk prediction.

## Methods

### Study population

We used data from a subset population from the China Cardiometabolic Disease and Cancer Cohort study. The details of this cohort have been described elsewhere (18, 19). In brief, 10999 individuals aged over 40 years from Tianmen City, Hubei province were enrolled in 2011. Health, lifestyle, and sociodemographic data were collected through questionnaires and interviews; participants also underwent a physical examination and provided blood samples. Written informed consent was obtained from each participant before the survey. Of the 10999 individuals, those with missing data regarding anthropometric measurements, blood pressure measurements or biochemical parameters were excluded. Moreover, as the METS-VF was derived from subjects with a BMI greater than 18.5 kg/m^2^, individuals with a BMI less than 18.5 were also excluded. Finally, 8866 individuals were included in this cross-sectional study.

### Central obesity assessment

Body weight was measured with a calibrated digital scale (Wuxi brand, RGZ120-RT) to the nearest 0.1 kg. Height was measured with a stadiometer to the nearest 0.1 cm without shoes. Waist circumference (WC) was measured at the midpoint between the last rib and iliac crest to the nearest 0.5 cm. The central obesity-related indices were calculated as follows:

(1) WHtR=WC (kg)/height (m^2^)

(2) METS-IR and METS-VF (17, 20)


$$METS-IR=\frac{Ln\left(\left(2\times G_{0}\right)+TG_{0}\right)\times BMI)}{Ln\left(HDL-C\right)}$$

$$\begin{array}{c} METS-VF=4.466+0.011\left[{\left(Ln\left(METS-IR\right)\right)}^{3}\right]\\ \;\;\;\;+3.239\left[{\left(Ln\left(WHtR\right)\right)}^{3}\right]+0.319\left(Sex\right)\\ \;\;\;\;+0.594(Ln\left(Age\right)), \end{array}$$

where G_0_ is expressed in mg/dL, TG_0_ in mg/dL, BMI in Kg/m^2^, HDL-C in mg/dL, Age in years, and sex was a binary response variable (men=1, women=0).

(3) LAP (21)


LAP(men)=(WC(cm)−65)×(TG(mmol/L))

LAP(women)=(WC(cm)−58)×(TG(mmol/L))

(4) VAI (22)


$$VAI\left(men\right)=\left(\frac{WC}{39.68+\left(1.88\times BMI\right)}\right)\times \left(\frac{TG}{1.03}\right)\times \left(\frac{1.31}{HDL-C}\right)$$

$$VAI\left(women\right)=\left(\frac{WC}{36.58+\left(1.89\times BMI\right)}\right)\times \left(\frac{TG}{0.81}\right)\times (\frac{1.52}{HDL-C}),$$

where WC is expressed in cm, BMI in Kg/m^2^, TG in mmol/L, and HDL in mmol/L.

(5) ABSI (23)


$$ABSI=\frac{WC}{BMI^{2/3}\times height^{1/2}},$$

where WC is expressed in m, BMI in Kg/m^2^, height in m.

(6) BRI (24)


$$BRI=364.2-365.5\sqrt{1-\frac{{\left(\frac{WC}{2\pi }\right)}^{2}}{{\left(0.5\times height\right)}^{2}}},$$

where WC is expressed in m, height in m.

(7) CMI (25)


$$CMI=\left(\frac{TG}{HDL-C}\right)\times WHtR,$$

where TG is expressed in mg/dL, HDL-C in mg/dL.

### Assessment of covariates and outcomes

Information on smoking habits, drinking habits, physical activity levels, and clinical history was collected through a standardized questionnaire. Current smoking was defined as smoking one or more cigarettes a day for at least six months. Current drinking was defined as having had one or more drinks of alcohol per week for at least six months. For physical activity, a metabolic equivalent (MET) value was assigned according to the compendium of activity energy costs for each activity in the questionnaire, and the total volume of physical activity was converted into MET-minutes per week (26); those who accumulated at least 600 MET-minutes of physical activity per week were classified as physically active. For medical history, CVD history was defined as having been diagnosed with myocardial infarction, coronary heart disease, stroke or peripheral artery disease.

Systolic and diastolic blood pressure were measured three times by using an Omron professional blood pressure monitor following a standardized protocol after the patients had been sitting for at least 5 minutes before measurement. Hypertension was defined as systolic blood pressure (SBP) ≥140 mmHg, diastolic blood pressure (DBP) ≥90 mmHg, or self-reported use of taking antihypertensive medications.

The 75g oral glucose tolerance test 75 was performed to evaluate the glucose metabolism status of the study participants. Venous fasting and 2-hour postload plasma glucose levels were measured by the enzymatic hexokinase method. HbA1c was measured by using a high-performance liquid chromatography method. Diabetes was defined as fasting blood glucose ≥7.0 mmol/L, 2-hour postload plasma glucose concentrations ≥11.1 mmol/L, HbA1c ≥6.5%, or self-reported diagnosis of diabetes and the use of glucose-lowering medications. Total, HDL, and LDL cholesterol, triglycerides, and serum creatinine were measured using fasting blood samples. eGFR was calculated on the basis of serum creatinine according to the Chronic Kidney Disease Epidemiology Collaboration (CKD-EPI) formula (27). CKD was defined as an eGFR<60 mL/min/1.73 m^2^.

### Statistical analysis

Normally distributed continuous variables are reported as the means (SDs), nonnormally distributed continuous variables are reported as median and interquartile ranges (IQRs). Categorical variables are presented as total numbers with corresponding percentages. Study population characteristics were compared between groups according to the presence of CKD. Differences between groups were evaluated by t test or one-way ANOVA for continuous variables and χ^2^ test for categorical variables. Associations of baseline METS-VF with CKD were assessed with logistic regression models, and odds ratios (ORs) and 95% confidence intervals (CIs) were calculated for participants in the highest tertile (T3) compared with participants in the two lower tertiles (T1–T2). Models were adjusted for current smoking, current drinking, physical activity level, diabetes, hypertension, CVD history and BMI. Stratified analyses by age (<60, ≥60 years), BMI (<24, ≥24), diabetes (no, yes), hypertension (no, yes) and history of CVD (no, yes) were also performed. Effect modification was tested by the likelihood ratio test comparing models with and without a multiplicative interaction term for the subgroup categories. Receiver operating characteristic (ROC) curve analysis was used to compare the predictive ability of these indices, and the areas under the ROC curve of different indices were compared using the method developed by DeLong et al. (28).The appropriate cut-off point of each index for the prediction of CKD was determined by using these indices as test variables and CKD as a state variable, and the optimal cut-off values were determined by maximizing the Youden index. All P values were two-sided and< 0.05 was considered statistically significant. The statistical analyses were performed using SPSS version 26.0 software (IBM Corporation, Chicago, IL) and R version 3.4.2 software.

## Results

### Baseline characteristics

Among the 8866 included participants, 35.2% were male, the mean (SD) age was 60.6 (10.1) years, and the mean eGFR was 94.4 (12.2) mL/min/1.73 m^2^. **Table 1** displays the baseline characteristics of all participants according to the presence of CKD. In the total population and among men and women, there was no significant difference in BMI between the CKD and non-CKD groups. However, the CKD group had significantly higher values for 8 central obesity indices (METS-VF, WC, WHtR, LAP, VAI, ABSI, BRI, and CMI) in the total population and among women; among men, the values for three (METS-VF, WHtR, and BRI) of the eight central obesity indices were higher in the CKD group than in the non-CKD group. At the same time, in the total population, the values for age, 2h-PG, HbA1c, TGs, HDL-C, and SBP and the proportions of individuals with hypertension, diabetes or a history of CVD were higher in the CKD group; among men, the values for 2h-PG and HDL-C and the proportion of individuals with a history of CVD were higher in the CKD group; among women, the values for HbA1c, TGs, HDL-C, and SBP and the proportions of individuals with hypertension or diabetes were higher in the CKD group.

**Table 1:** Participant characteristics of CKD and non-CKD populations.

	Total (n = 8866)			Men (n = 3117)			Women (n = 5749)		
	eGFR ≥60 (n=8707)	eGFR<60 (n=159)	*P* value	eGFR ≥60 (n=3056)	eGFR<60 (n=61)	*P* value	eGFR ≥60 (n=5651)	eGFR<60 (n=98)	*P* value
Age (years)	60.46 ± 10.04	68.32 ± 10.08	<0.001	62.42 ± 9.80	68.05 ± 8.33	<0.001	59.40 ± 10.00	68.49 ± 11.06	<0.001
Current smoker (%)	13.9	9.4	0.105	38.4	23.0	0.014	0.7	1.0	0.490
Current drinker (%)	12.5	3.1	<0.001	33.1	6.6	<0.001	1.4	1.0	0.999
Physically active (%)	62.9	55.3	0.051	61.8	54.1	0.218	63.5	56.1	0.135
FPG (mmol/L)	5.53 ± 1.28	5.84 ± 2.02	0.059	5.53 ± 1.10	5.68 ± 1.34	0.301	5.53 ± 1.37	5.93 ± 2.34	0.092
2-h PG (mmol/L)	6.92 ± 5.02	7.83 ± 3.65	0.023	6.69 ± 2.59	7.45 ± 3.29	0.024	7.04 ± 5.93	8.07 ± 3.86	0.089
HbA1c (%)	5.81 ± 0.87	6.01 ± 1.03	0.004	5.73 ± 0.68	5.90 ± 0.94	0.051	5.85 ± 0.95	6.07 ± 1.08	0.023
TGs (mmol/L)	1.23 (0.89-1.77)	1.39 (1.02-1.92)	0.005	1.10 (0.81-1.64)	1.16 (0.96-1.58)	0.254	1.30 (0.95-1.82)	1.51 (1.16-2.12)	0.002
HDL-C (mmol/L)	1.50 ± 0.36	1.41 ± 0.33	0.002	1.52 ± 0.40	1.41 ± 0.31	0.044	1.50 ± 0.33	1.42 ± 0.34	0.016
SBP (mmHg)	150.62 ± 23.83	157.97 ± 26.37	<0.001	152.82 ± 23.51	155.93 ± 24.47	0.307	149.44 ± 23.92	159.23 ± 27.53	0.001
DBP (mmHg)	81.68 ± 12.40	82.45 ± 15.52	0.533	82.82 ± 13.38	84.36 ± 17.73	0.503	81.05 ± 11.79	81.26 ± 13.94	0.886
Hypertension (%)	68.1	81.8	<0.001	72.2	82.0	0.092	65.9	81.6	0.001
Diabetes (%)	12.5	19.5	0.009	12.0	16.4	0.294	12.8	21.4	0.012
CVD (%)	7.3	11.9	0.027	8.4	19.7	0.002	6.7	7.1	0.875
METS-VF	6.40 ± 0.59	6.66 ± 0.53	<0.001	6.52 ± 0.58	6.75 ± 0.55	0.001	6.34 ± 0.58	6.60 ± 0.51	<0.001
LAP	24.32 (13.92-41.60)	29.83 (17.02-53.60)	0.002	18.25 (9.90-32.76)	22.25 (12.32-33.91)	0.129	27.96 (16.68-46.00)	37.92 (21.54-59.55)	0.001
VAI	1.38 (0.86-2.25)	1.61 (1.00-2.70)	0.003	0.93 (0.61-1.58)	1.18 (0.74-1.54)	0.099	1.63 (1.08-2.55)	2.16 (1.41-3.27)	<0.001
ABSI	0.078 ± 0.007	0.081 ± 0.007	<0.001	0.078 ± 0.007	0.079 ± 0.005	0.237	0.078 ± 0.007	0.081 ± 0.007	<0.001
BRI	3.65 ± 1.17	4.04 ± 1.24	<0.001	3.40 ± 1.03	3.73 ± 1.03	0.012	3.79 ± 1.21	4.24 ± 1.33	<0.001
CMI	0.97 (0.63-1.59)	1.22 (0.73-1.95)	<0.001	0.86 (0.54-1.47)	1.07 (0.68-1.54)	0.058	1.04 (0.68-1.65)	1.39 (0.91-2.09)	<0.001
WHtR	0.51 ± 0.06	0.53 ± 0.06	<0.001	0.50 ± 0.05	0.52 ± 0.05	0.01	0.52 ± 0.06	0.54 ± 0.06	<0.001
WC (cm)	81.15 ± 9.20	83.47 ± 9.33	0.002	82.32 ± 9.07	84.18 ± 8.53	0.112	80.51 ± 9.21	83.03 ± 9.80	0.007
BMI (kg/m^2^)	23.70 ± 3.09	23.86 ± 3.34	0.527	23.43 ± 2.99	24.01 ± 3.25	0.137	23.85 ± 3.13	23.77 ± 3.41	0.798

Continuous variables are expressed as the means (standard deviations) or medians (IQRs), and categorical variables are expressed as numbers (percentages). 2-h PG, 2-hour postprandial blood glucose; TGs, triglycerides; TC, total cholesterol; HDL-C, high-density lipoprotein cholesterol; LDL-C, low-density lipoprotein cholesterol; SBP, systolic blood pressure; DBP, diastolic blood pressure; CVD, cardiovascular disease; METS-VF, metabolic score for visceral fat; LAP, lipid accumulation product; VAI, visceral adiposity index; ABSI, a body shape index; BRI, body roundness index; CMI, cardiometabolic index; WHtR, waist-to-height ratio; WC, waist circumference; BMI, body mass index.

### Associations of the METS-VF with CKD risk

**Table 2** shows the associations between the METS-VF and CKD. Positive associations were found between the METS-VF and the prevalence of CKD in the overall population and the population stratified by sex. When unadjusted (Model 1), the participants in the highest tertile of the METS-VF had a significantly higher risk of CKD than participants in the lowest tertile of the METS-VF (OR 2.838, 95% CI 1.888-4.264 for the total population; OR 3.063, 95% CI 1.539-6.093 for men; OR 3.058, 95% CI 1.787-5.233 for women). After adjusting for current smoking, current drinking, and physical activity (Model 2), the ORs showed little change. In Model 3, which was additionally adjusted for hypertension, diabetes, and history of CVD, the ORs in the highest tertile vs. the lowest tertile were as follows: 2.489 (1.635-3.789) for the overall population, 2.805 (1.389-5.663) for men, 2.585 (1.487-4.495) for women. In Model 4, we further adjusted for BMI and found that the OR value for the highest tertile vs. the lowest tertile increased; the corresponding ORs were 3.943 (2.435-6.385) for the overall population, 3.585 (1.585-8.109) for men, 4.627 (2.485-8.616) for women. In the fully adjusted model (Model 4), each SD increase in the METS-VF was associated with a 110.2% higher risk of CKD in the overall population, a 76.1% higher risk of CKD among men and with a 130.1% higher risk of CKD among women.

**Table 2:** Associations between METS-VF and CKD.

	METS-VF tertiles			P for trend	Per 1 SD increase
	Tertile 1	Tertile 2	Tertile 3		
Total					
Median (range)	5.84 (≤6.22)	6.48 (6.23-6.71)	6.97 (>6.71)		
Cases, n (%)	32 (1.1%)	38 (1.3%)	89 (3.0%)	<0.001	
Model 1	1	1.190 (0.742-1.910)	2.838 (1.888-4.264)	<0.001	1.689 (1.402-2.035)
Model 2	1	1.206 (0.751-1.937)	2.912 (1.930-4.393)	<0.001	1.699 (1.406-2.053)
Model 3	1	1.139 (0.708-1.832)	2.489 (1.635-3.789)	<0.001	1.568 (1.293-1.902)
Model 4	1	1.397 (0.857-2.275)	3.943 (2.435-6.385)	<0.001	2.102 (1.653-2.674)
Men					
Median (range)	5.99 (≤6.32)	6.58 (6.33-6.83)	7.07 (>6.83)		
Cases, n (%)	11 (1.1%)	17 (1.6%)	33 (3.2%)	<0.001	
Model 1	1	1.551 (0.723-3.329)	3.063 (1.539-6.093)	0.001	1.627 (1.208-2.192)
Model 2	1	1.567 (0.728-3.373)	3.100 (1.550-6.200)	0.001	1.613 (1.199-2.169)
Model 3	1	1.540 (0.714-3.322)	2.805 (1.389-5.663)	0.003	1.527 (1.132-2.060)
Model 4	1	1.698 (0.773-3.730)	3.585 (1.585-8.109)	0.002	1.761 (1.205-2.573)
Women					
Median (range)	5.78 (≤6.16)	6.42 (6.17-6.65)	6.90 (>6.65)		
Cases, n (%)	18 (0.9%)	26 (1.4%)	54 (2.8%)	<0.001	
Model 1	1	1.450 (0.792-2.653)	3.058 (1.787-5.233)	<0.001	1.717 (1.350-2.184)
Model 2	1	1.460 (0.798-2.672)	3.141 (1.833-5.380)	<0.001	1.740 (1.366-2.215)
Model 3	1	1.340 (0.730-2.460)	2.585 (1.487-4.495)	<0.001	1.582 (1.235-2.026)
Model 4	1	1.747 (0.937-3.254)	4.627 (2.485-8.616)	<0.001	2.301 (1.692-3.128)

Model 1: Unadjusted.

Model 2: Adjusted for sex (only in total population), current smoking, current drinking, and physical activity.

Model 3: Adjusted for sex (only in total population), current smoking, current drinking, physical activity, hypertension, diabetes, and CVD.

Model 4: Adjusted for sex (only in total population), current smoking, current drinking, physical activity, hypertension, diabetes, CVD and BMI.

### Subgroup analyses for the association between the METS-VF and CKD risk

In the subgroup analyses (**Table 3**), significant interactions were found between the METS-VF and risk of CKD by age (P value for interaction = 0.023). The association appeared to be significantly stronger among those aged over 60 years than younger individuals when comparing individuals in tertile 3 vs. tertile 1. When stratified by other factors, comparing individuals in tertile 3 vs. tertile 1, the association between METS-VF and risk of CKD was more pronounced among individuals with overweight/obesity, individuals without diabetes, individuals without hypertension, and individuals without CVD history. However, none of these interaction terms reached statistical significance.

**Table 3:** Subgroup analysis of the association between METS-VF and CKD.

	METS-VF tertiles			P for trend	P for interaction
Subgroup	Tertile 1	Tertile 2	Tertile 3		
Age (years)					
<60	1	2.073 (0.759-5.663)	4.880 (1.403-16.980)	0.015	0.023
≥60	1	1.062 (0.605-1.866)	2.492 (1.450-4.282)	0.001	
BMI					
Normal	1	1.434 (0.852-2.411)	3.339 (1.966-5.671)	<0.001	0.779
Overweight/Obesity	1	1.171 (0.251-5.459)	3.877 (0.931-16.142)	0.001	
Diabetes					
No	1	1.273 (0.739-2.194)	4.036 (2.381-6.839)	<0.001	0.359
Yes	1	1.786 (0.571-5.594)	3.255 (0.990-10.702)	0.048	
Hypertension					
No	1	2.252 (0.765-6.632)	14.578 (5.172-41.091)	<0.001	0.926
Yes	1	1.221 (0.707-2.108)	2.815 (1.641-4.831)	<0.001	
CVD history					
No	1	1.436 (0.852-2.420)	4.445 (2.665-7.413)	<0.001	0.847
Yes	1	1.260 (0.317-5.005)	1.479 (0.340-6.427)	0.601	

Adjusted for sex, current smoking, current drinking, physical activity, hypertension, diabetes, CVD and BMI, except for the stratifying factor.

### ROC analyses of the METS-VF and other central obesity indices with CKD risk

**Figure 1:**
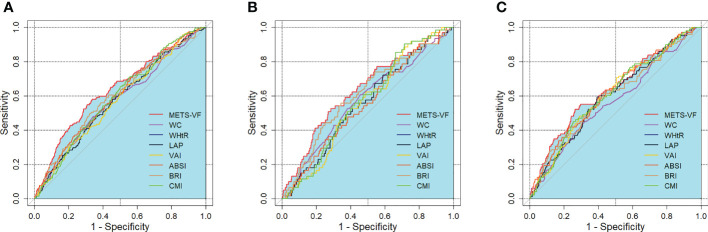
ROC curves for the prediction of CKD by adiposity indices. **(A)** total population, **(B)** men, **(C)** women. METS-VF, metabolic score for visceral fat; LAP, lipid accumulation product; VAI, visceral adiposity index; ABSI, a body shape index; BRI, body roundness index; CMI, cardiometabolic index; WHtR, waist-to-height ratio; WC, waist circumference; BMI, body mass index.

**Table 4** and **Figure 1** show the AUC scores (and 95% CIs) for the 8 central obesity indices for predicting CKD risk for total population and for both sexes. In the total population, both the METS-VF and seven other central obesity indices could identify CKD. The METS-VF had a higher AUC value than that of WC, WHtR, LAP, VAI and BRI and had a comparable value to that of ABSI and CMI. Among men, the METS-VF, WHtR, and BRI could identify CKD. The METS-VF had a higher AUC value than that of WHtR and BRI. Among women, all 8 indices could identify CKD. The METS-VF had a higher AUC value than that of WC, WHtR and BRI and a comparable value to that of LAP, VAI, ABSI and CMI.

**Table 4:** ROC analyses for the prediction of CKD by adiposity indices.

	AUC	95% CI	p value	Cut-off value	Sensitivity (%)	Specificity (%)	Youden index
Total							
METS-VF	0.634	0.589-0.680	<0.001	6.705	57.23	66.85	24.08
WC	0.571*	0.525-0.617	0.002	85.000	47.80	65.92	13.72
WHtR	0.595*	0.550-0.640	<0.001	0.535	49.69	67.64	17.33
LAP	0.572*	0.528-0.616	0.002	25.520	59.75	52.46	12.21
VAI	0.569*	0.525-0.612	0.003	1.295	66.04	46.56	12.60
ABSI	0.596	0.552-0.639	<0.001	0.077	70.44	44.02	14.46
BRI	0.595*	0.550-0.640	<0.001	4.041	49.69	67.64	17.33
CMI	0.589	0.547-0.631	<0.001	0.892	70.44	44.95	15.39
Men							
METS-VF	0.632	0.559-0.704	<0.001	6.891	52.46	71.47	23.93
WC	0.573	0.500-0.646	0.051	–	–	–	–
WHtR	0.605*	0.533-0.677	0.005	0.519	55.74	65.67	21.41
LAP	0.557	0.489-0.624	0.129	–	–	–	–
VAI	0.562	0.500-0.623	0.099	–	–	–	–
ABSI	0.555	0.487-0.623	0.144	–	–	–	–
BRI	0.605*	0.533-0.677	0.005	3.713	55.74	65.67	21.41
CMI	0.571	0.509-0.633	0.058	–	–	–	–
Women							
METS-VF	0.634	0.577-0.692	<0.001	6.744	52.04	73.47	25.51
WC	0.568*	0.509-0.626	0.021	85.000	44.90	68.11	13.01
WHtR	0.598*	0.539-0.657	0.001	0.525	61.22	57.10	18.32
LAP	0.599	0.543-0.655	0.001	33.920	59.18	60.77	19.95
VAI	0.608	0.553-0.663	<0.001	1.610	70.41	49.39	19.80
ABSI	0.619	0.563-0.675	<0.001	0.083	41.84	77.07	18.91
BRI	0.598*	0.539-0.657	0.001	3.846	61.22	57.10	18.32
CMI	0.610	0.555-0.665	<0.001	1.641	44.90	74.55	19.45

*P< 0.05 when comparing the AUC with METS-VF.

AUC, area under the curve; CI, confidence interval; METS-VF, metabolic score for visceral fat; LAP, lipid accumulation product; VAI, visceral adiposity index; ABSI, a body shape index; BRI, body roundness index; CMI, cardiometabolic index; WHtR, waist-to-height ratio; WC, waist circumference; BMI, body mass index.

In the total population, the optimal cut-off values were 6.705 for the METS-VF, 85.000 for WC, 0.535 for WHtR, 25.520 for LAP, 1.295 for the VAI, 0.077 for ABSI, 4.041 for BRI, and 0.892 for CMI. For men, the optimal cut-off values were 6.891 for the METS-VF, 0.519 for WHtR, and 3.713 for BRI. For women, the optimal cut-off values were 6.744 for the METS-VF, 85.000 for WC, 0.525 for WHtR, 33.920 for LAP, 1.610 for the VAI, 0.083 for ABSI, 3.846 for BRI, and 1.641 for CMI. In the total population and for both sexes, the METS-VF had the highest Youden index values for identifying CKD.

## Discussion

In this cross-sectional study, we found that the METS-VF was positively associated with the risk of CKD, and this association was independent of current smoking, current drinking, physical activity, diabetes, hypertension, history of CKD and BMI. Moreover, the METS-VF is a good indicator for CKD compared with other central obesity indices, including WC, WHtR, LAP, VAI, ABSI, BRI, and CMI. The METS-VF showed better predictive ability for CKD among men and better or comparable predictive ability for CKD in the total population and among women. In the total population and for both sexes, the METS-VF had the highest Youden index.

The impact of visceral adiposity on kidney function has been evaluated by several studies. For example, Kang et al. used the multifrequency bioelectrical impedance analysis method to measure visceral body fat and found that a higher level of visceral adiposity was associated with a higher prevalence of CKD, and the association remained significant after adjusting for age, sex, diabetes, and hypertension (29). Visceral adiposity has also been associated with kidney disease progression. For example, Hiroshi et al. measured visceral fat and subcutaneous fat area by CT scan, and reported that the visceral-to-subcutaneous fat ratio was longitudinally associated with the risk of a more than 30% decline in eGFR among individuals with established CKD (30). These studies used bioelectrical impedance methods or imaging methods to measure visceral adiposity, which provide high measurement accuracy and effectively proved the role of excessive visceral adiposity in the pathogenesis or the progression of CKD. However, these methods are often not feasible in population-based epidemiological studies. Thus, using anthropometric indicators in CKD risk prediction is essential.

Waist circumference has long been used to assess central obesity and related disease risk; however, for individuals with similar WCs, WC may overestimate the risk for tall people and underestimate the risk for short people (31). To overcome the shortcomings of WC, indices including WHtR, ABSI and BRI adjusted for weight and/or BMI in their formulas. Moreover, the VAI, LAP and CMI integrate lipid parameters into their formulas, which enable them not only to assess of the mass of adipose tissue but also to reflect the dysfunction of adipose tissue. In our study, we focused on the newly invented-METS-VF index. The METS-VF algorithm mainly consists of three parts: an insulin resistance component (METS-IR), an anthropometric component (WHtR) and a demographic component (age, sex). The METS-IR component can reflect the degree of insulin resistance, and metabolic dysregulation in central obesity, which play critical roles in the pathogenesis of CKD. Moreover, the METS-VF was reported to have the ability to provide relatively accurate measurements of visceral adiposity and insulin resistance even in metabolically healthy obese individuals who do not have substantial laboratory disturbances (17). Meanwhile, the METS-VF has been demonstrated to be a strong predictor for hypertension and diabetes in Western and Chinese populations, and has stronger predictive power than several of the abovementioned indices (17, 32, 33). Overall, these characteristics and advantages of the METS-VF may facilitate its use in CKD risk prediction.

Through logistic analysis and ROC analysis, we proved the acceptable predictive ability of the METS-VF in CKD risk assessment. Moreover, we noted several points in exploring the relationship between the MRTS-VF and CKD in logistic analysis. First, sex differences were noted when we additionally adjusted for BMI in Model 4, which should be explained. The METS-VF is a measurement for visceral adipose tissue; for a given METS-VF, a higher BMI value may indicate elevations in lower body subcutaneous adipose tissue and muscle mass. It has been reported that BMI is positively correlated with visceral adipose tissue mass measured by magnetic resonance imaging when not adjusted for WC, but negatively correlated with visceral adipose tissue mass after adjusting for WC; thus, when assessing the association between central obesity and health outcomes, the strength of the association may not be fully realized until after adjustment for BMI (34, 35). In this study, after adjusting for BMI, the association was more evident among women than among men. Sex differences in fat distribution may partially account for this phenomenon; men tend to have relatively more visceral fat, while women have relatively more subcutaneous fat. The hyperplasia of subcutaneous adipose tissue can provide safe storage of excess lipids and reduce the spillover of excess lipids to visceral adipose tissue or other normally lean organs, contributing the maintenance of a metabolic health phenotype (36). Thus, subcutaneous tissue may modulate the association between the METS-VF and CKD risk to a higher degree among women than among men. Second, we found that sex can modify the association between the METS-VF and CKD risk. Among individuals aged less than 60 years, the association was significantly stronger, and the reasons behind this still need further investigation. In this study, the proportion of individuals with diabetes, hypertension and a history of CVD was significantly higher among those aged more than 60 years. Although our data did not include the duration of these comorbidities, it is likely that older individuals would have had these comorbidities for a longer period of time and that the kidneys would have tended to have more exposure to these risk factors, which might influence the relationship between visceral fat and CKD when setting these comorbidities as confounders.

Currently, a series of studies aimed at determining the best adiposity indices for predicting CKD in the Chinese population have been published. Several new indices, such as the VAI and LAP, were evaluated, as they have been reported to be better indicators for cardiovascular diseases or events than traditional central obesity indices such as WC and WHtR (22, 37). Dai et al. reported that the VAI and LAP were superior to BMI, WHtR and WC in identifying CKD as defined by an estimated GFR< 60 ml/min/1.73m^2^ for men but not for women aged more than 35 years (38). Chen et al. reported that the VAI had better discriminative ability for CKD defined by an estimated GFR< 60 ml/min/1.73m^2^ or the presence of albuminuria than BMI and WC for women but not for men aged 50–90 years (39). In this study, we took the VAI and LAP into consideration when comparing the predictive performance of the METS-VF with other indices. Three other novel visceral adiposity indices (ABSI, BRI, CMI) were also considered, as they were reported be linked with CKD in other ethnic groups (40) or with other cardiometabolic diseases in the Chinese population (41). We found that the METS-VF had the best predictive power for CKD among all indices for men, as the METS-VF had the highest AUC value in the analysis. For women, its performance was also acceptable, as it had higher or similar AUC values than other indices. Thus, our study makes important contributions to the literature on this topic.

Our study has several strengths. First, this is the first study to explore the association between the METS-VF and CKD and compare its performance with several traditional central obesity indices. Second, this was a community population-based study with a relatively large sample size, and the results can be representative of the general population. Third, the use of a standardized protocol for anthropometric measurement guarantees the accuracy of the study results. However, our study has several limitations. First, the cross-sectional design could not provide an interpretation of the causation or directionality of the association. Second, CKD was defined on the basis of a single measurement of eGFR, and the presence of microalbuminuria was not included, as we did not collect urine specimens from the participants. Third, as physical activity is associated with the risk of incident CKD and CKD related outcomes (42, 43), we included physical activity levels as a confounder in the logistic analysis. However, it is not easy to objectively measure population-level physical activity levels and we used self-reported data, which may be subjected to recall bias. Fourth, the included participants in this study were aged more than 40 years and recruited from a Chinese population; thus, the generalizability of our findings to individuals younger than 40 years or of other ethnicities remains to be verified.

## Conclusions

In conclusion, our study demonstrated that the METS-VF is closely associated with the risk of CKD after adjusting for potential confounders. Moreover, we found that the METS-VF has a superior ability to predict CKD than other indices (WC, WHtR, LAP, VAI, ABSI, BRI, CMI), and its advantage was particularly pronounced for men. The optimal cut-off values for the METS-VF in predicting CKD are 6.891 for men and 6.744 for women. The significant relationship between the METS-VF and the risk of CKD has important public health implications. This reminds us that in health management work, we should attach importance to visceral obesity in individuals at high risk of CKD, and interventions to reduce visceral adiposity should be adopted in CKD prevention.

## Data availability statement

The datasets analysed in the current study are not publicly available due to the limits on the data-sharing agreement of the China Cardiometabolic Disease and Cancer Cohort study group.

## Ethics statement

The study protocol was approved by the medical ethics committee of Ruijin Hospital, Shanghai Jiao Tong University. The patients/participants provided their written informed consent to participate in this study.

## Author contributions

PY, XY contributed to the study conception and study design. PY performed the data analysis; PY, XM, RK and ZW interpreted the data; PY wrote the manuscript. All authors read and approved the final manuscript.

## Funding

This research was funded by grants from the National Natural Science Foundation of China (82270880, 81570740).

## Acknowledgments

We thank all the staff and participants of China Cardiometabolic Disease and Cancer Cohort Study for their great efforts in the survey.

## Conflict of interest

The authors declare that the research was conducted in the absence of any commercial or financial relationships that could be construed as a potential conflict of interest.

## Publisher’s note

All claims expressed in this article are solely those of the authors and do not necessarily represent those of their affiliated organizations, or those of the publisher, the editors and the reviewers. Any product that may be evaluated in this article, or claim that may be made by its manufacturer, is not guaranteed or endorsed by the publisher.
